# Transcriptome Analysis Reveals That NEFA and β-Hydroxybutyrate Induce Oxidative Stress and Inflammatory Response in Bovine Mammary Epithelial Cells

**DOI:** 10.3390/metabo12111060

**Published:** 2022-11-02

**Authors:** Chengmin Li, Junpeng Huang, Xiangxing Chen, Yexiao Yan, Lian Li, Weiguo Zhao

**Affiliations:** 1 School of Biotechnology, Jiangsu University of Science and Technology, Zhenjiang 212100, China; 2 Zibo Service Center for Animal Husbandry and Fishery, Zibo 255000, China; 3 College of Animal Science and Technology, Nanjing Agricultural University, Nanjing 210095, China

**Keywords:** non-esterified fatty acids, β-hydroxybutyrate, oxidative stress, inflammatory responses, bovine mammary epithelial cells

## Abstract

Non-esterified fatty acids (NEFA) and β-hydroxybutyrate (BHBA) are the metabolites of fat mobilization initiated by negative energy balance (NEB) during the perinatal period in dairy cows, which have an adverse effect on cell physiology of various bovine cell types. The aim of this study was to explore the biological roles of NEFA and BHBA on provoking oxidative stress and inflammatory responses in bovine mammary epithelial cells (BMECs). RNA sequencing analysis showed that there are 1343, 48, and 1725 significantly differentially expressed genes (DEGs) in BMECs treated with NEFA, BHBA and their combination. GO functional analysis revealed that the DEGs were significantly enriched in “response to oxidative stress” and “inflammatory response”. Further study demonstrated that NEFA and BHBA elevated the malondialdehyde (MDA) and reactive oxygen species (ROS) accumulation and reduced the total superoxide dismutase (T-SOD) and glutathione peroxidase (GSH-Px) activity to cause oxidative stress. In addition, expression of inflammatory markers (NO, TNF-α, IL-6, and IL-1β) were increased after NEFA and BHBA stimulation. Mechanistically, our data showed that NEFA and BHBA activated the MAPK signaling pathway. Collectively, our results indicate that NEFA and BHBA induce oxidative stress and inflammatory response probably via the MAPK signaling pathway in BMECs.

## 1. Introduction

Transition dairy cows, from late gestation to early lactation, are more likely to experience metabolic changes (ketosis, fatty liver) and infectious (metritis, mastitis) diseases [1,2]. During the transition period, cows generally undergo a state of negative energy balance (NEB) which could be responsible for the above physiologic challenges [3,4]. Severe NEB triggered lipid mobilization and a subsequent high circulating concentration of non-esterified fatty acids (NEFA), as well as β-hydroxybutyrate (BHBA). Clinical data from previous studies indicated that elevated concentrations of NEFA and BHBA were associated with the greater incidences of postpartum diseases (mastitis, ketosis, clinical endometritis, or metritis, as well as other conditions correlated with immunosuppression), which have adverse effects on general health, longevity, and productive and reproductive performances of dairy cows [5,6,7,8].

NEFA could act as signaling and energy-rich molecules and have an adverse effect on the cell physiology of various bovine cell types. Previous research confirmed that high concentrations of NEFA could induce apoptotic damage and inflammation in bovine granulosa cells and hepatocytes [9,10,11,12], hamper bovine oviductal epithelial cell physiology [13], and compromise oocyte maturation and early embryo physiology [14]. Moreover, results from our other previous studies indicated that NEFA treatment induced ER stress, apoptosis, inflammation, and oxidative stress in bovine mammary epithelial cells (BMECs) [15,16,17]. Similarly, BHBA also had a cytotoxicity effect on multiple bovine cell types, negative effects of BHBA have been shown in hepatocytes [18], endometrial cells [19], granulosa cells [20], and mammary epithelial cells [21]. Particularly, BHBA can also function as signal molecule by activating pathways involved in inflammation and oxidative stress [18,19,22].

During negative energy balance, high plasma concentrations of NEFA and BHBA can be absorbed by the mammary gland, thereby augmenting the response of mammary epithelium to metabolic stress. BMECs participate in the first line of mammary gland defense against external stimulus. Previous studies have found that NEFA could induce inflammatory response and oxidative stress in BMECs [15,17]. However, the exact molecular mechanism involved in such processes are yet to be fully illustrated, and until now, the biological roles of BHBA and its combinative effect with NEFA on triggering oxidative and inflammatory reactions have not been explored. Thus, the aim of this study was to investigate the potential molecular mechanisms of NEFA, BHBA, and their combinative effects on provoking oxidative stress and inflammatory responses in BMECs.

## 2. Materials and Methods

### 2.1. Cell Culture and Treatments

Bovine mammary epithelial cell line (BMECs, MAC-T) were a gift from Dr. Youping Sun. Cells were cultured in DMEM/F12 medium (Gibco, Grand Island, NY, USA) supplemented with 10% FBS (Zeta life, Menlo Park, CA, USA) and 200 U/mL of penicillin and streptomycin (Invitrogen, Carlsbad, CA, USA), and incubated at 37 °C in an atmosphere of 90% humidity and 5% CO_2_. The medium was changed every 48 h. 

BMECs were treated with NEFA (0.9 mM, 6 h), BHBA (2.4 mM, 24 h), and their combination [16,19]. Before the treatment, cells were starved overnight in FBS-free DMEM/F12 medium. The stock solution of NEFA was 50 mM, containing 15.95 mM palmitic acid, 21.75 mM oleic acid, 2.65 mM palmitoleic acid, 2.45 mM linoleic acid, and 7.2 mM stearic acid.

### 2.2. Assay for Malondialdehyde, Superoxide Dismutase, and Glutathione Peroxidase

The levels of total superoxide dismutase (T-SOD), malondialdehyde (MDA), and glutathione peroxidase (GSH-Px) were measured in the supernatant of BMECs using a biochemical analysis kit (Nanjing Jiancheng Bioengineering Institute, Nanjing, China) according to the manufacturer’s protocol. The optical density of MDA, SOD, and GSH-Px was determined by a microplate reader at absorption wavelengths of 532 nm, 450 nm, and 412 nm, respectively.

### 2.3. Intracellular Reactive Oxygen Species Measurement

The BMECs were seeded into six-well plates at 1 × 10^6^ cells and then treated with NEFA, BHBA, and their combination. The intracellular level of ROS was determined by a corresponding commercial kit (Nanjing, China). The method was based on the ROS-sensitive probe DCFH-DA, which can enter cells freely through living cell membranes and be hydrolyzed into DCFH by intracellular esterases, and DCFH is easily oxidized to DCF (dichlorofluorescein) which is a strong green fluorescent substance that can be measured at excitation and emission wavelengths of 500 and 525 nm. BMECs were incubated with 10 µM of DCFH-DA in the dark for 30 min at 37 °C, then resuspended in PBS and immediately analyzed by a fluorescence microscope (Zeiss LSM 700 META (Olympus, Tokyo, Japan)). ROS fluorescence intensity was quantified by Image J software (National Institutes of Health, Bethesda, MD, USA).

### 2.4. Nitric Oxide Staining Assay

The level of nitric oxide (NO) was measured by a corresponding commercial kit (Beyotime, Beijing, China), according to the manufacturer’s instructions. After NEFA and BHBA treatment, BMECs were incubated with the fluorescent probe DAF-FM DA at 37 °C for 30 min, then washed in PBS and analyzed by a fluorescence microscope (Zeiss LSM 700 META (Olympus, Tokyo, Japan)).

### 2.5. Enzyme-Linked Immunosorbent Assay (ELISA)

The cells were treated as described above. The supernatant levels of tumor necrosis factor-α (TNF-α), interleukin (IL)-6, and IL-1β were measured using a commercially available enzyme-linked immunosorbent assay kit (MIBio, Shanghai, China) according to the manufacturer’s instructions. The results were measured spectrophotometrically at a wavelength of 450 nm.

### 2.6. Real-Time Quantitative PCR Analysis

TRIzol reagent (Invitrogen, Carlsbad, CA, USA) were used to extract total RNA of the BMECs. The purity and quantity of total RNA were detected by NanoDrop 1000 spectrophotometer (Thermo Scientific, Wilmington, DE, USA). DNase I and the Prime Script RT Master Mix kit (TaKaRa, Otsu, Japan) were used for DNA removal and cDNA synthesis, respectively. Real-time quantitative PCR was performed using SYBR ^®^Premix Ex Taq™ (TaKaRa, Otsu, Japan) on an Applied Biosystem 7500 HT Sequence detection system. The PCR program consisted of one cycle at 95 °C for 30 s, 40 cycles at 95 °C for 5 s, and 60 °C for 34 s, and fluorescence signal collection at 60 °C. All the primers used in this study were shown in Table S1. Data were normalized to glyceraldehyde-3-phosphate dehydrogenase (GAPDH), the gene expression levels were calculated using 2^–ΔΔCt^ method.

### 2.7. RNA-Seq and Transcriptome Analysis

Three samples were selected from the control, NEFA, BHBA, and their combination treatment group for acquiring transcriptome sequences. RNA extraction, quality control, and sequencing were performed as described previously [16]. Reads were filtered by fastp (v 0.18.0) and mapped to the reference genome (Bos Taurus, assembly ARS-UCD1.2 (GCA_002263795.2), http://asia.ensembl.org/Bos_taurus/Info/Index (accessed on 5 May 2021) using HISAT2. 2.4. The mapped reads of each sample were assembled by using StringTie (v1.3.1) in a reference-based approach. DESeq2 software were used for RNAs differential expression analysis. Differentially expressed genes (DEGs) were identified based on the parameter of false discovery rate (FDR) below 0.05 and absolute fold change ≥2. Hypergeometric tests were used to evaluate the enrichment score. A *p*-value ≤ 0.05 indicated significantly enriched GO terms and KEGG and Reactome pathway. The raw data were submitted to the National Center for Biotechnology Information (NCBI) under BioProject accession number PRJNA869860 and PRJNA880749. 

### 2.8. Western Blot Analysis

BMECs were harvested after NEFA and BHBA treatment. The total BMECs protein were extracted and quantified as described previously [16]. Sodium dodecyl sulfate-polyacrylamide gel electrophoresis (SDS-PAGE) was performed to separate the equal amounts of proteins, fluoride (PVDF) membranes were used to transfer the protein from gel to membrane. The membranes were then incubated in blocking solution and probed with the following primary antibodies: rabbit anti-SOD2 (1:1000, 24127-1-AP, Proteintech, Chicago, IL, USA), rabbit anti-p38 MAPK (A10832), phosphor-p38 MAPK-Y182 (AP0057), ERK (A16686), phosphor-ERK1-T202/Y204+ERK2-T185/Y187 (AP1120), JNK (A18678), and phosphor-JNK-T183/Y185 (AP1163) (1:1000, ABclonal, Boston, MA, USA), and rabbit anti- Tubulin (1:1000, Bioworld, Louis Park, MN, USA). Then, the blots were washed three times with TBST and incubated with a secondary antibody. ImageJ software (National Institutes of Health, Bethesda, MD, USA) were used to quantify the immunoblots.

### 2.9. Statistical Analysis

GraphPad Prism 6.01 software (GraphPad Software Inc., San Diego, CA, USA) was used for statistical analyses. All values are shown as the mean ± standard error of the mean (SEM) from three repeats of each experiment that was run in triplicate. The comparisons of more than two groups were determined using one-way analysis of variance (ANOVA) followed by Tukey’s test. A Student’s *t* test was used to compare the two sets of data.

## 3. Results

### 3.1. Expression Dynamics of mRNAs in BMECs under the Treatment with NEFA and BHBA

**Figure 1:**
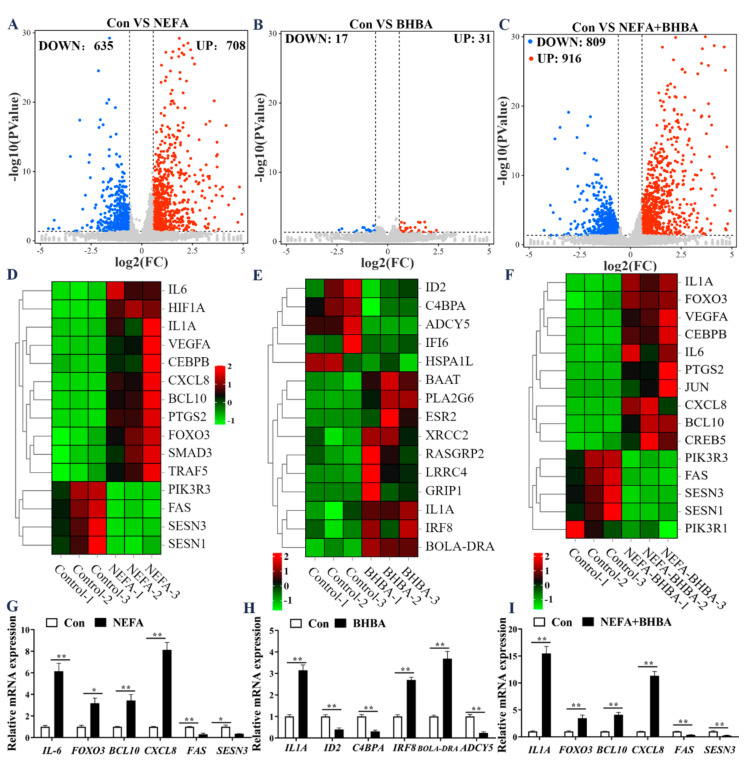
DEG analysis of BMECs treated with NEFA and BHBA. (**A**) Volcano of DEGs between control and NEFA treatment group in BMECs. (**B**) Volcano of DEGs between control and BHBA treatment group in BMECs. (**C**) Volcano of DEGs between control and combinative treatment (NEFA and BHBA) group in BMECs. (**D**–**F**) Heatmaps of DEGs involved in oxidative stress and inflammatory response in BMECs. (**G**–**I**) qPCR verification of the EDGs in BMECs treated with NEFA and BHBA. Data are presented as the means ± SEM of three independent experiments. * *p* < 0.05; ** *p* < 0.01.

To clarify the cytotoxic molecular mechanisms of NEFA and BHBA in the BMECs, transcriptome sequencing and analysis were performed. Analysis of DEGs under NEFA treatment caused a total of 1343 DEGs, of which 708 genes were upregulated and 635 genes were downregulated (Figure 1A). Following BHBA treatment, 31 genes were upregulated, and 17 genes were downregulated (Figure 1B). Furthermore, combinative treatment of NEFA and BHBA resulted in 916 upregulated genes and 809 downregulated genes (Figure 1C). Significantly, the enrichment results of DEGs contained oxidative stress (FOXO3, SESN1, SESN3, etc.) and inflammatory response-related (IL-6, CXCL8, BCL10, etc.) genes, the expression patterns of these genes in each sequencing group are shown in Figure 1D–F. Six DEGs of each group were selected for verification by qPCR and the results showed that these genes have similar expression dynamics as RNA-seq data (Figure 1G–I).

### 3.2. GO Enrichment Analysis of DEGs in BMECs under the Treatment with NEFA and BHBA Formatting of Mathematical Components

**Figure 2:**
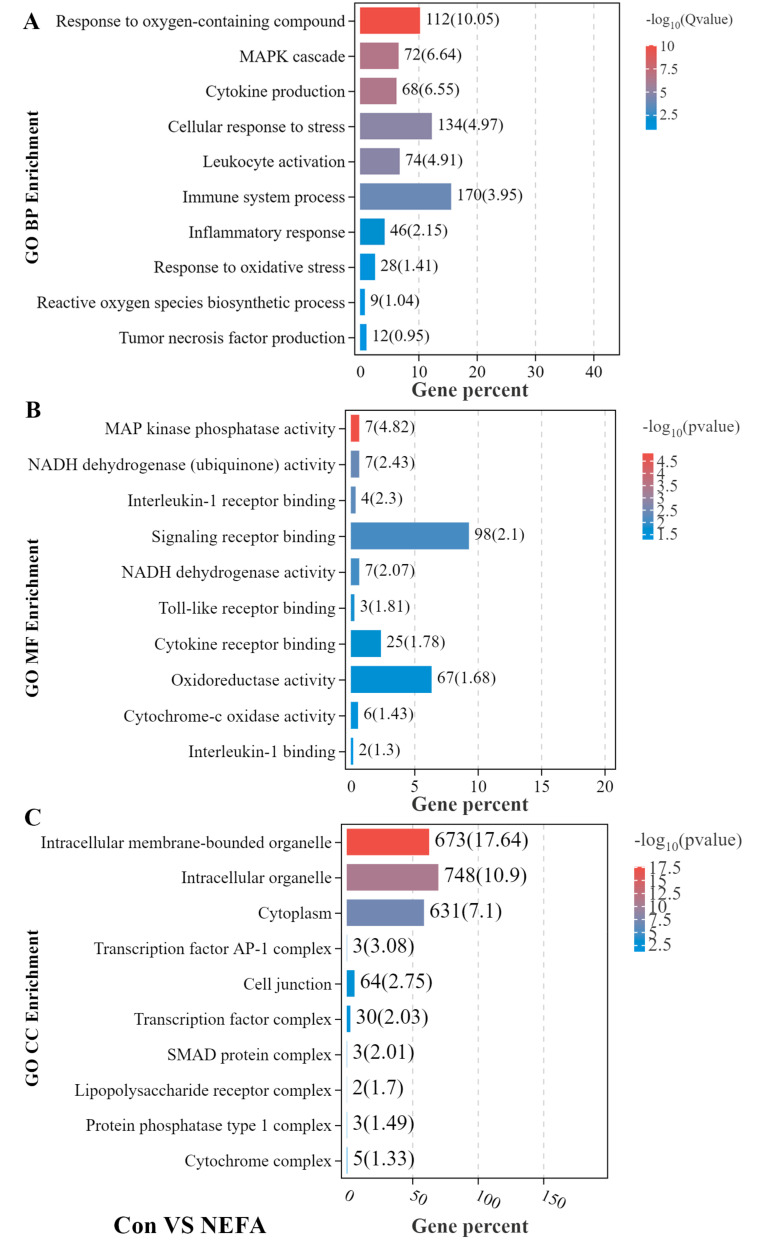

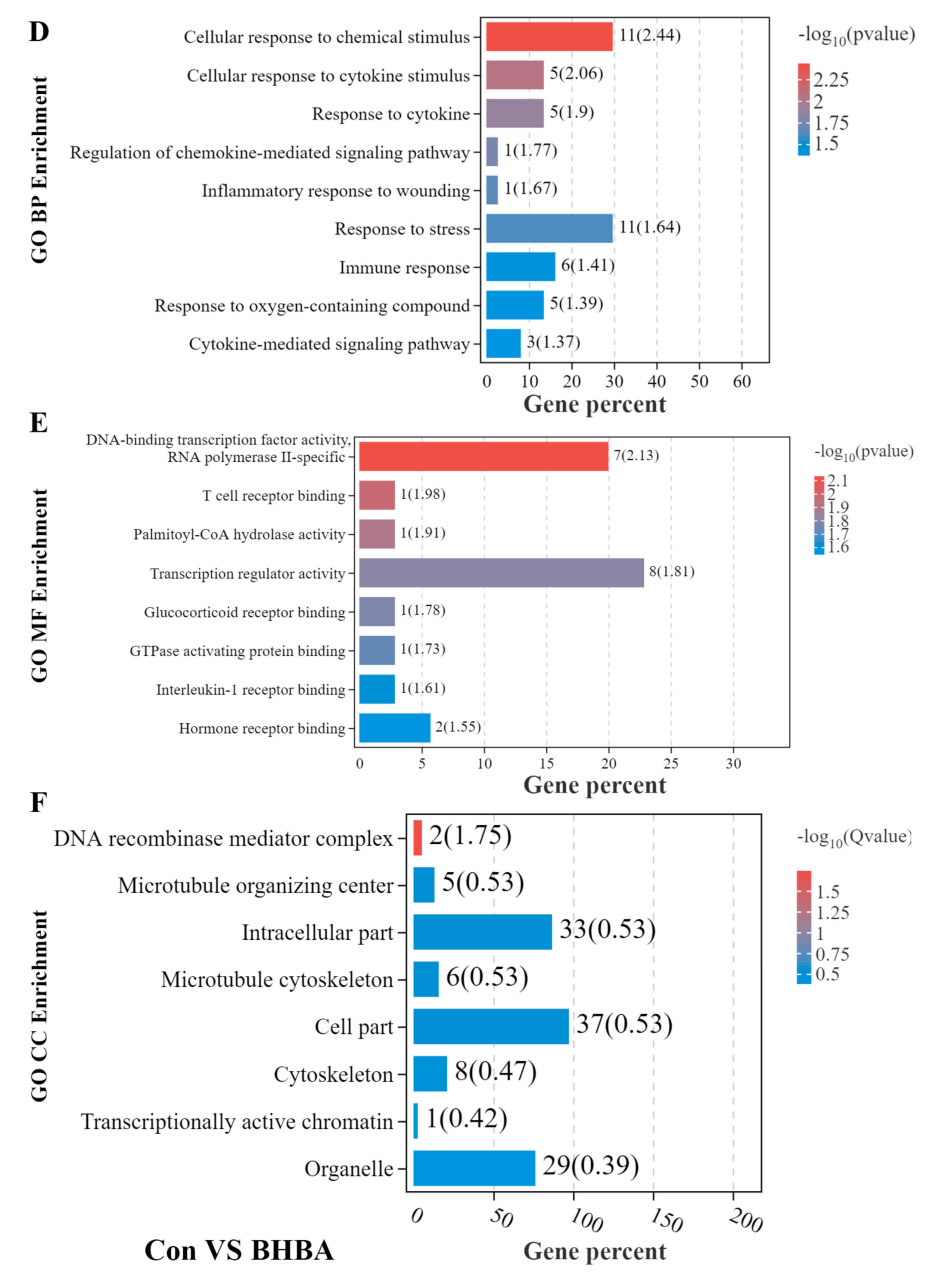

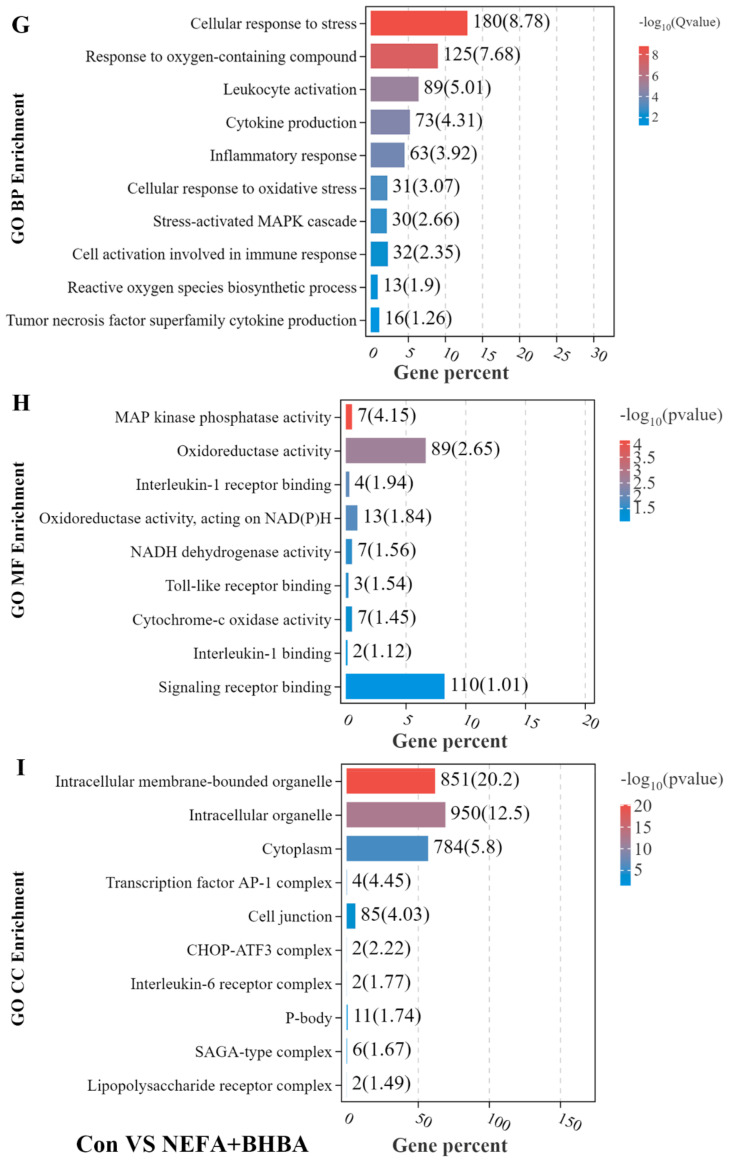
GO functional enrichment analysis of DEGs. (**A**–**C**) GO enrichment analysis of DEGs between control and NEFA treatment group. (**D**−**F**) GO enrichment analysis of DEGs between control and BHBA treatment group. (**G**–**I**) GO enrichment analysis of DEGs between control and the combinative treatment (NEFA and BHBA) group in BMECs. BP, biological processes; MF, molecular function; CC, cellular component.

As shown in Figure 2A,D,G, response to oxidative stress, inflammatory response, leukocyte activation, and response to cytokine and cytokine production were significantly enriched in the biological processes. Regarding the molecular function, we found that oxidoreductase activity, cytokine receptor binding, and interleukin-1 binding were significantly enriched (Figure 2B,E,H). Furthermore, the significant cellular component included intracellular organelle, lipopolysaccharide receptor complex (Figure 2C,F,I). Collectively, GO function analysis data revealed that oxidative stress and inflammatory response could be induced by NEFA, BHBA, and their combination treatment.

### 3.3. NEFA and BHBA Treatment Induced Oxidative Stress in BMECs

**Figure 3:**
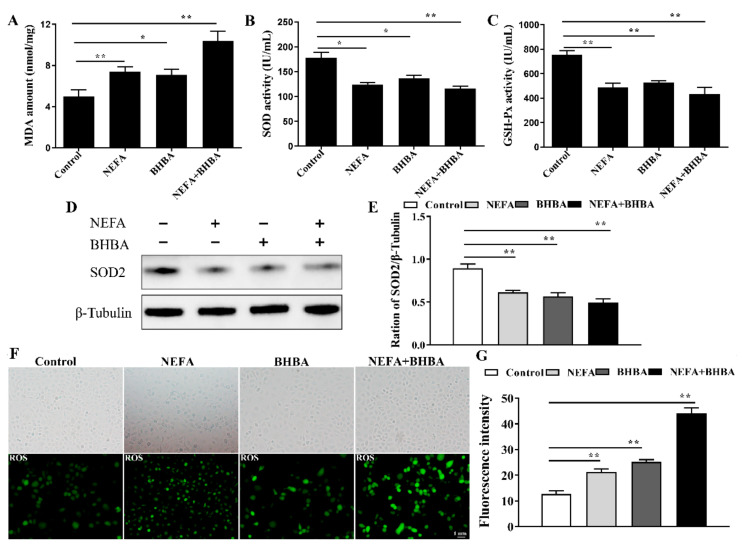
NEFA and BHBA treatment induced oxidative stress in BMECs. (**A**–**C**) levels of MDA, SOD, and GSH-Px in BMECs treated with NEFA and BHBA. (**D**,**E**) Protein expression of SOD2 in BMECs treated with NEFA and BHBA. (**F**) ROS levels in BMECs treated with NEFA and BHBA. (**G**) Green fluorescence intensity of NO was quantified by Image J. Data are presented as the means ± SEM of three independent experiments. * *p* < 0.05; ** *p* < 0.01.

Based on the GO enrichment analysis, we further assessed the role of NEFA and BHBA on inducing oxidative stress in BMECs. As shown in Figure 3A–C, compared with the control group, NEFA, BHBA, and their combination treatment significantly increased the content of MDA and decreased the levels of SOD and GSH-Px. Consistent with the biochemical data, Western blotting analysis showed that the SOD2 protein level was markedly decreased under stimulation with NEFA, BHBA and their combination (Figure 3D). Meanwhile, fluorescent staining showed that ROS signals were higher in the treatment group than that in the control group (Figure 3F). These results validated that oxidative stress was activated in BMECs during the NEFA, BHBA, and their combination treatment.

### 3.4. NEFA and BHBA Treatment Induced Inflammatory Response in BMECs

**Figure 4:**
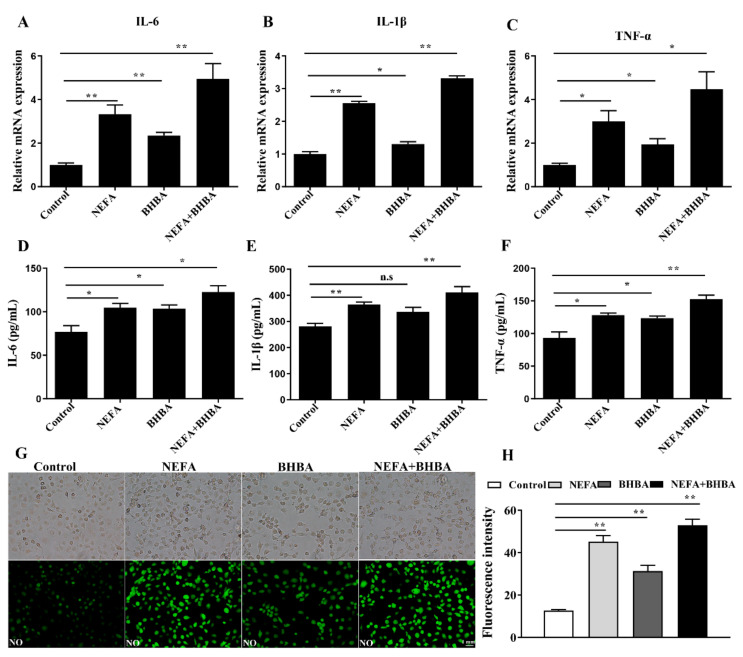
NEFA and BHBA treatment induced inflammatory response in BMECs. (**A**–**C**) qPCR analysis of TNF-α, IL-1β, and IL-6 in BMECs treated with NEFA and BHBA. (**D**–**F**) ELISA analysis of IL-1β, IL-6, and TNF-α in BMECs treated with NEFA and BHBA. (**G**) Immunofluorescence staining of NO in BMECs. (**H**) Green fluorescence intensity of NO was quantified by Image J. Data are presented as the means ± SEM of three independent experiments. * *p* < 0.05; ** *p* < 0.01; n.s. means not significant.

The effect of NEFA and BHBA on inducing inflammatory response in BMECs was also further analyzed. Results showed that the level of proinflammatory cytokines (IL-6, TNF-α, and IL-1β) was considerably increased under stimulation with NEFA, BHBA, and their combination when compared with the control group (Figure 4A–F). Similarly, NEFA and BHBA treatment also significantly increased the levels of inflammatory mediator NO (Figure 4G).

### 3.5. MAPK Signaling Pathway Was Involved in NEFA and BHBA-Induced Oxidative Stress and Inflammatory Response

**Figure 5:**
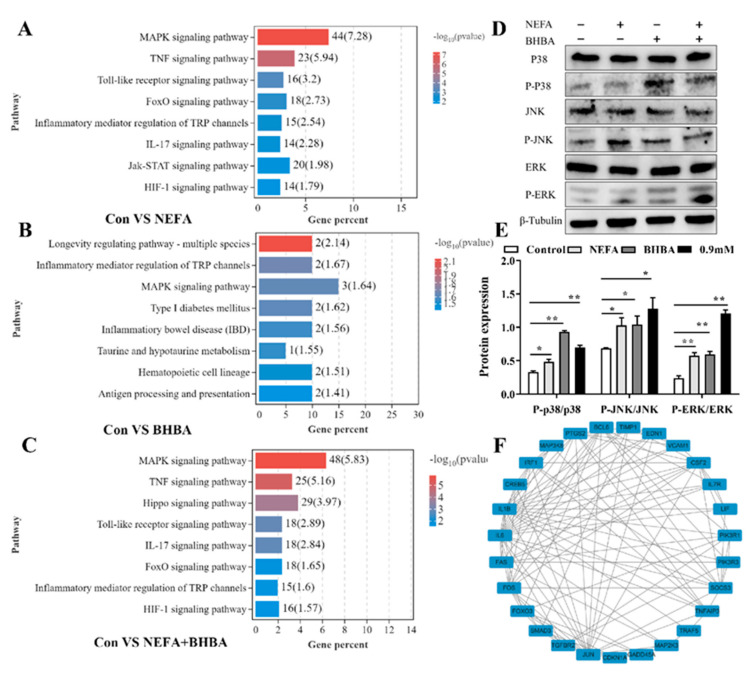
MAPK Pathway was activated by NEFA and BHBA. (**A**–**C**) KEGG enrichment analysis of DEGs in BMECs treated with NEFA and BHBA. (**D**,**E**) Protein expression of MAPK pathway-related genes in BMECs treated with NEFA and BHBA. (**F**) PPI network of differentially expressed genes selected in the MAPK and TNF signaling pathway. Data are presented as the means ± SEM of three independent experiments. * *p* < 0.05; ** *p* < 0.01.

According to the KEGG pathway analysis, the regulation of MAPK signaling pathway may be the most likely underlying mechanism of oxidative stress and inflammation induced by NEFA and BHBA in BMECs (Figure 5A–C). The MAPK pathway-related protein expression levels were determined by Western blotting; results showed that the phosphorylated p38 MAPK, c-Jun N-terminal kinase (JNK), and extracellular signal-regulated kinase (ERK) expression were significantly upregulated in the treatment groups compared with the control group (Figure 5D). In addition, the PPI network of differentially expressed genes selected in the MAPK and TNF signaling pathway was constructed using the STRING website (Figure 5F); these differentially expressed genes were involved in oxidative stress (FOXO3, SMAD3, BCL6, etc.) and inflammation (IL-1β, IL-6, FAS, JUN, etc.) processes. Taken together, these data revealed that NEFA, BHBA, and their combination treatment predominantly activated the MAPK pathway.

## 4. Discussion

In the present study, we studied the molecular response of BMECs to high concentrations of NEFA and BHBA, with a focus on oxidative stress and inflammatory response. The experimental results here demonstrated that NEFA, BHBA, and their combination treatment (1) induced oxidative stress, as indicated by increased MDA and ROS accumulation and decreased SOD and GSH-Px activities, (2) induced the inflammatory reactions, characterized by significant elevation of cytokine expression, and (3) activated the MAPK signaling pathway. These findings illustrated the effects and common molecular events of NEFA and BHBA on triggering oxidative stress and inflammatory response in BMECs.

High plasma concentrations of NEFA and BHBA during the time cows face the challenge of NEB could be loaded and absorbed by the mammary gland, thereby inducing a state of oxidative stress [22,23]. Oxidative stress, caused by an imbalance between pro-oxidants and antioxidants, is mainly characterized by excessive ROS accumulation. High levels of ROS have been demonstrated to be closely related to pathological processes in vitro, resulting in substantial damage to cells and organelles by oxidation of cellular lipids, proteins, and DNA [24,25]. Based on our RNA-seq results, differences in oxidative stress between NEFA and BHBA treatment and control were huge (Figure 2). Work in in vitro experiments demonstrated that exogenous NEFA and BHBA could increase ROS generation, which subsequently leads to oxidative stress in multiple bovine cell types [17,26,27]. In our present study, higher ROS and MDA level coupled with lower activities of GSH-Px and SOD (Figure 3) proved that NEFA and BHBA induced oxidative stress in mammary epithelial cells. These findings are consistent with the previous experiment.

Studies showed that oxidative stress and inflammatory response are closely connected and could synergistically contribute to metabolic and infectious disorders in perinatal dairy cows; oxidative stress-mediated inflammation has long been identified as triggers of miscellaneous inflammatory diseases [1,28,29,30]. Peripheral blood levels of IL-1B, IL-6, and TNF-a were higher in NEB cows than in healthy transition cows [31]. Moreover, previous research by Chankeaw et al. found that elevated NEFA promoted cytokine production and induced inflammatory response in bovine hepatocytes and endometrial epithelial cells [12,32]. Moreover, it is indicated that BHBA could also cause inflammatory responses in various cell types of transition cows via activating NF-κB and AMPK signaling [27,33]. In the present study, the GO and KEGG enrichment analysis suggest that NEFA, BHBA-related DEGs were significantly enriched in inflammatory reaction (Figure 1, Figure 2 and Figure 5). We also found that BMECs exhibited higher levels of TNF-α, IL-6, IL-1β, and NO after NEFA, BHBA, and their combination treatment compared to those in the control group (Figure 4), which was consistent with the results obtained in other bovine cell types. These findings suggest that NEFA and BHBA can act as proinflammatory factors to induce inflammation in BMECs.

The MAPK pathways, represented by p38MAPK, JNK, and ERK, are activated by a diverse array of extra- and intracellular stimuli including inflammatory cytokines and oxidative stress [34,35]. Previously published data showed that MAPK signaling pathway was connected with the metabolic process and immune response in transition cows [36]. An in vitro experiment in research by Song et al. showed that BHBA stimulation activated the p38 MAPK pathway and resulted in bovine hepatocyte apoptosis [37]. Furthermore, our recent previous study demonstrated that MAPK signaling pathway is involved in ER stress-mediated apoptosis in NEFA-treated BMECs [16]. Consistent with these results, we found that NEFA, BHBA, and their combination activated the MAPK signaling pathway in bovine mammary epithelial cells, suggesting that MAPK pathway probably mediated the oxidative stress and inflammation induced by NEFA and BHBA in BMECs.

## 5. Conclusions

**Figure 6:**
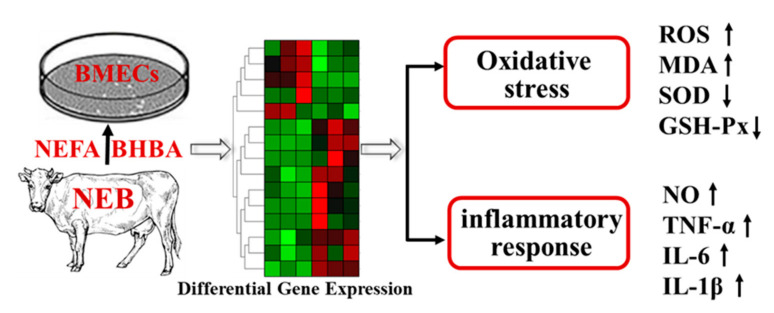
Schematic plot for the toxicity of NEFA and BHBA to BMECs. NEFA and BHBA induces oxidative stress (increased MDA and ROS levels, decreased SOD and GSH-Px activities) and inflammatory response (elevated cytokine expression) in BMECs.

In summary, our results provide new knowledge on the role of NEFA and BHBA in oxidative stress and inflammatory response of bovine mammary epithelial cells. In the current study, both NEFA and BHBA induced oxidative damage and inflammation, and their combination is endowed with a synergistic effect (Figure 6). Further analysis demonstrated that MAPK signaling pathway was activated and probably contributed to the NEFA and BHBA-induced oxidative stress and inflammation in BMECs. These findings provide a new basis for exploring the prevention and treatment of metabolic and infectious diseases in dairy cows with an NEB.

## Data Availability

The authors confirmed that all data underlying the findings are fully available, the relevant data are within the paper.

## Supplementary Materials

The following supporting information can be downloaded at: https://www.mdpi.com/article/10.3390/metabo12111060/s1, Table S1: Primers sequences for real-time quantitative PCR analysis.

## References

[1] Kuhla B. (2020). Review: Pro-inflammatory cytokines and hypothalamic inflammation: Implications for insufficient feed intake of transition dairy cows. Animal.

[2] Goselink R.M.A., Schonewille J.T., van Duinkerken G., Hendriks W.H. (2020). Physical exercise prepartum to support metabolic adaptation in the transition period of dairy cattle: A proof of concept. J. Anim. Physiol. Anim. Nutr..

[3] Ospina P.A., Nydam D.V., Stokol T., Overton T.R. (2010). Evaluation of nonesterified fatty acids and beta-hydroxybutyrate in transition dairy cattle in the northeastern United States: Critical thresholds for prediction of clinical diseases. J. Dairy Sci..

[4] Pascottini O.B., Leroy J., Opsomer G. (2022). Maladaptation to the transition period and consequences on fertility of dairy cows. Reprod. Domest. Anim..

[5] Ospina P.A., McArt J.A., Overton T.R., Stokol T., Nydam D.V. (2013). Using nonesterified fatty acids and β-hydroxybutyrate concentrations during the transition period for herd-level monitoring of increased risk of disease and decreased reproductive and milking performance. Vet. Clin. N. Am. Food Anim. Pract..

[6] Chapinal N., Carson M.E., LeBlanc S.J., Leslie K.E., Godden S., Capel M., Santos J.E., Overton M.W., Duffield T.F. (2012). The association of serum metabolites in the transition period with milk production and early-lactation reproductive performance. J. Dairy Sci..

[7] McArt J.A., Nydam D.V., Oetzel G.R., Overton T.R., Ospina P.A. (2013). Elevated non-esterified fatty acids and β-hydroxybutyrate and their association with transition dairy cow performance. Vet. J..

[8] Benedet A., Costa A., De Marchi M., Penasa M. (2020). Heritability estimates of predicted blood β-hydroxybutyrate and nonesterified fatty acids and relationships with milk traits in early-lactation Holstein cows. J. Dairy Sci..

[9] Wang Y., Li C., Ali I., Li L., Wang G. (2020). N-acetylcysteine modulates non-esterified fatty acid-induced pyroptosis and inflammation in granulosa cells. Mol. Immunol..

[10] Wang Y., Li C., Li J., Wang G., Li L. (2020). Non-Esterified Fatty Acid-Induced Reactive Oxygen Species Mediated Granulosa Cells Apoptosis Is Regulated by Nrf2/p53 Signaling Pathway. Antioxidants.

[11] Song Y., Li X., Li Y., Li N., Shi X., Ding H., Zhang Y., Li X., Liu G., Wang Z. (2014). Non-esterified fatty acids activate the ROS-p38-p53/Nrf2 signaling pathway to induce bovine hepatocyte apoptosis in vitro. Apoptosis.

[12] Shi X., Li D., Deng Q., Li Y., Sun G., Yuan X., Song Y., Wang Z., Li X., Li X. (2015). NEFAs activate the oxidative stress-mediated NF-κB signaling pathway to induce inflammatory response in calf hepatocytes. J. Steroid Biochem. Mol. Biol..

[13] Jordaens L., Arias-Alvarez M., Pintelon I., Thys S., Valckx S., Dezhkam Y., Bols P.E., Leroy J.L. (2015). Elevated non-esterified fatty acid concentrations hamper bovine oviductal epithelial cell physiology in three different in vitro culture systems. Theriogenology.

[14] Aardema H., van Tol H.T.A., Vos P. (2019). An overview on how cumulus cells interact with the oocyte in a condition with elevated NEFA levels in dairy cows. Anim. Reprod. Sci..

[15] Liu L., Lu H., Loor J.J., Aboragah A., Du X., He J., Peng T., Su J., Wang Z., Liu G. (2021). Sirtuin 3 inhibits nuclear factor-κB signaling activated by a fatty acid challenge in bovine mammary epithelial cells. J. Dairy Sci..

[16] Yan Y., Huang J., Huan C., Li L., Li C. (2022). Non-Esterified Fatty Acid Induces ER Stress-Mediated Apoptosis via ROS/MAPK Signaling Pathway in Bovine Mammary Epithelial Cells. Metabolites.

[17] Chen Y., Tang Y., Luo S., Jia H., Xu Q., Chang R., Dong Z., Gao S., Song Q., Dong H. (2021). Nuclear factor erythroid 2-related factor 2 protects bovine mammary epithelial cells against free fatty acid-induced mitochondrial dysfunction in vitro. J. Dairy Sci..

[18] Lei L., Gao W., Loor J.J., Aboragah A., Fang Z., Du X., Zhang M., Song Y., Liu G., Li X. (2021). Reducing hepatic endoplasmic reticulum stress ameliorates the impairment in insulin signaling induced by high levels of β-hydroxybutyrate in bovine hepatocytes. J. Dairy Sci..

[19] Cheng X., Yang S., Xu C., Li L., Zhang Y., Guo Y., Zhang C., Li P., Long M., He J. (2019). Proanthocyanidins Protect against β-Hydroxybutyrate-Induced Oxidative Damage in Bovine Endometrial Cells. Molecules.

[20] Gong J., Zhao S., Heng N., Wang Y., Hu Z., Wang H., Zhu H. (2022). The Dynamic Transcription Profiles of Proliferating Bovine Ovarian Granulosa When Exposed to Increased Levels of β-Hydroxybutyric Acid. Front. Vet. Sci..

[21] Hillreiner M., Flinspach C., Pfaffl M.W., Kliem H. (2016). Effect of the Ketone Body Beta-Hydroxybutyrate on the Innate Defense Capability of Primary Bovine Mammary Epithelial Cells. PLoS ONE.

[22] Song Y., Loor J.J., Li C., Liang Y., Li N., Shu X., Yang Y., Feng X., Du X., Wang Z. (2021). Enhanced mitochondrial dysfunction and oxidative stress in the mammary gland of cows with clinical ketosis. J. Dairy Sci..

[23] Kuroiwa T., Matsuda K., Kanazawa T., Chee H., Kimura A., Satoh H., Sato S., Ichijo T. (2022). Effect of Oxidative Status on the Occurrence of Haemolactia in Dairy Cows after Calving. J. Vet. Res..

[24] Schieber M., Chandel N.S. (2014). ROS function in redox signaling and oxidative stress. Curr. Biol..

[25] Sun X., Jia H., Xu Q., Zhao C., Xu C. (2019). Lycopene alleviates H_2_O_2_-induced oxidative stress, inflammation and apoptosis in bovine mammary epithelial cells via the NFE2L2 signaling pathway. Food Funct..

[26] Chang R., Sun X., Jia H., Xu Q., Dong Z., Tang Y., Luo S., Jiang Q., Loor J.J., Xu C. (2022). Inhibiting nuclear factor erythroid 2 related factor 2-mediated autophagy in bovine mammary epithelial cells induces oxidative stress in response to exogenous fatty acids. J. Anim. Sci. Biotechnol..

[27] Li P., Li L., Zhang C., Cheng X., Zhang Y., Guo Y., Long M., Yang S., He J. (2019). Palmitic Acid and β-Hydroxybutyrate Induce Inflammatory Responses in Bovine Endometrial Cells by Activating Oxidative Stress-Mediated NF-κB Signaling. Molecules.

[28] Bernabucci U., Ronchi B., Lacetera N., Nardone A. (2005). Influence of body condition score on relationships between metabolic status and oxidative stress in periparturient dairy cows. J. Dairy Sci..

[29] Bradford B.J., Yuan K., Farney J.K., Mamedova L.K., Carpenter A.J. (2015). Invited review: Inflammation during the transition to lactation: New adventures with an old flame. J. Dairy Sci..

[30] Castillo C., Hernandez J., Bravo A., Lopez-Alonso M., Pereira V., Benedito J.L. (2005). Oxidative status during late pregnancy and early lactation in dairy cows. Vet. J..

[31] Contreras G.A., Sordillo L.M. (2011). Lipid mobilization and inflammatory responses during the transition period of dairy cows. Comp. Immunol. Microbiol. Infect. Dis..

[32] Chankeaw W., Guo Y.Z., Båge R., Svensson A., Andersson G., Humblot P. (2018). Elevated non-esterified fatty acids impair survival and promote lipid accumulation and pro-inflammatory cytokine production in bovine endometrial epithelial cells. Reprod. Fertil. Dev..

[33] Xu T., Lu X., Arbab A.A.I., Wu X., Mao Y., Loor J.J., Yang Z. (2021). Metformin acts to suppress β-hydroxybutyric acid-mediated inflammatory responses through activation of AMPK signaling in bovine hepatocytes. J. Anim. Sci..

[34] Hotamisligil G.S., Davis R.J. (2016). Cell Signaling and Stress Responses. Cold Spring Harb. Perspect. Biol..

[35] Kim E.K., Choi E.J. (2010). Pathological roles of MAPK signaling pathways in human diseases. Biochim. Biophys. Acta.

[36] Veshkini A., Hammon H.M., Lazzari B., Vogel L., Gnott M., Tröscher A., Vendramin V., Sadri H., Sauerwein H., Ceciliani F. (2022). Investigating circulating miRNA in transition dairy cows: What miRNAomics tells about metabolic adaptation. Front. Genet..

[37] Song Y., Li N., Gu J., Fu S., Peng Z., Zhao C., Zhang Y., Li X., Wang Z., Li X. (2016). β-Hydroxybutyrate induces bovine hepatocyte apoptosis via an ROS-p38 signaling pathway. J. Dairy Sci..

