# Supplementary material for: Transcriptome Analysis Reveals That NEFA and β-Hydroxybutyrate Induce Oxidative Stress and Inflammatory Response in Bovine Mammary Epithelial Cells
Source: Metabolites. 2022 Nov 2;12(11):1060. doi: 10.3390/metabo12111060 (PMC9696823; doi:10.3390/metabo12111060)
Supplement: Supplementary file 1 [file metabolites-12-01060-s001.zip › metabolites-1981891-supplementary.pdf]

Table S1. Primers sequences for real-time quantitative PCR analysis.

Gene	Accession number	Primer sequences (5′-3′)	fragment size (bp)
IL1A	NM_174092.1	F:CATAGACGGCAGCCCACCAG	232
		R: AGACCCCGGGAGAAGGCAAT	
FOXO3	XM_005210804.4	F: CAGGGCAAAGTCAGGTGCCA	165
		R: ACCCTGCCTCGTCTGACTGT	
BCL10	NM_001078028.1	F: GGAGGCGCCACAGTTTGACT	169
		R: CGGGAGATGGCGGTTCTTCC	
CXCL8	NM_173925.2	F: TGGCTGTTGCTCTCTTGGCA	256
		R: TGCACAACCTTCTGCACCCA	
FAS	NM_174662.2	F: CGCCCAGTCATGTCCGACTC	101
		R: AGAAGGCAATGGCACCCCAC	
SESN3	XM_024975719.1	F: TTGCCAGACGAGGGGAGGAA	196
		R: CTGCGCAGCATCGTTGTGTC	
ID2	NM_001034231.2	F: GCTTCCCTCCTCCCAATCGC	200
		R: CTGGGCACCAGCTCCTTGAG	
C4BPA	NM_174252.3	F: AAGGAGGCACGCCTGAAAGG	133
		R: AGAGAAGGGCCAGGATGCCA	
IRF8	NM_001083769.2	F: GGCTACGCTGCGCTTTGAAC	178
		R: TCGCCCACACTCCATCTCCA	
BOLA-DRA	NM_001012677.1	F: TGCCCTGGCCAATATGGCTG	183
		R: ACACTGATCACGGGTGGGGA	
ADCY5	NM_001192069.1	F: GCGCAACGACGAGCTCTACT	243
		R: GAGTCATTGAGGCCGGAGGC	
TNF-α	NM_173966.3	F: CGACATCAACTCTCCGGGGC	198
		R: GCAATGCGGCTGATGGTGTG	
IL-6	NM_173923.2	F: CGCTTCACAAGCGCCTTCAC	249
		R: TGCCAGTGTCTCCTTGCTGC	
IL-1β	NM_174093.1	F: ACAGCCATGGCAACCGTACC	219
		R: CATGGCCACGATGACCGACA	
β-Actin	NM_173979.3	F: GGGCAGGTCATCACCATCGG	244
		R: TCATTGTGCTGGGTGCCAGG	
